# Concomitant Legionella pneumophila and Streptococcus pneumoniae Infections With Refractory Necrotizing Pneumonia in a Patient Receiving Immunosuppressive Therapy for COVID-19

**DOI:** 10.7759/cureus.92531

**Published:** 2025-09-17

**Authors:** Shunsuke Fukuda, Atsushi Nakahira, Naoyuki Shiraishi, Koichi Maeda, Kazuaki Atagi

**Affiliations:** 1 Department of Critical Care Medicine, Nara Prefecture General Medical Center, Nara, JPN; 2 Department of Infectious Diseases, Nara Prefecture General Medical Center, Nara, JPN

**Keywords:** baricitinib, concomitant bacterial pneumonia, covid-19, extracorporeal membrane oxygenation, immunosuppressive therapy, legionella pneumophila, necrotizing pneumonia, refractory pneumonia, streptococcus pneumoniae, the elderly

## Abstract

Bacterial co-infection in patients with coronavirus disease 2019 (COVID-19) is uncommon but represents a clinically significant complication. Such infections present both diagnostic and therapeutic challenges and are often associated with poor outcomes. We report a rare case of refractory necrotizing pneumonia caused by co-infection with *Legionella pneumophila* and *Streptococcus pneumoniae* in a patient with COVID-19 who was treated with dexamethasone and baricitinib. This case underscores the importance of maintaining a high index of suspicion for concomitant bacterial infections in immunosuppressed elderly patients with COVID-19 to ensure timely and appropriate intervention and thereby improve clinical outcomes.

## Introduction

Bacterial infection has been documented to concurrently occur in 7% of patients with coronavirus disease 19 (COVID-19) at the time of their initial visit to healthcare facilities and also has an impact on their mortality [1]. Furthermore, a multicenter international observational study reported that approximately 54% of patients with COVID-19 who were admitted to an intensive care unit setting suffered from concomitant infection [2]. However, only a few cases are known to have multiple bacterial co-infections during the treatment course of COVID-19 [3]. We present a case of an elderly individual with immunosuppressive therapy for COVID-19, who developed co-infection with *Legionella pneumophila* and *Streptococcus pneumoniae,* resulting in refractory necrotizing pneumonia, along with the findings from microbiological autopsy.

## Case presentation

A 76-year-old man with a history of emphysema, who was independent in activities of daily living, presented with a sore throat and tested positive for COVID-19. He had no history of diabetes mellitus or other conditions suggestive of immunocompromise. Two days after the onset of symptoms, he was admitted to a nearby hospital due to dyspnea and was started on remdesivir, baricitinib, and intravenous dexamethasone at a dose of 7.6 mg once daily, with a diagnosis of worsening COVID-19 pneumonia. On the fifth day of admission, the chest radiograph revealed decreased aeration in the left lower lung, prompting the initiation of ceftriaxone in conjunction with the suspicion of concomitant bacterial pneumonia. On the ninth day of admission, he exhibited exacerbating dyspnea and hypoxemia despite the escalation of antibiotic treatment to tazobactam/piperacillin and he was transferred to our hospital for intensive care treatment.

**Figure 1:**
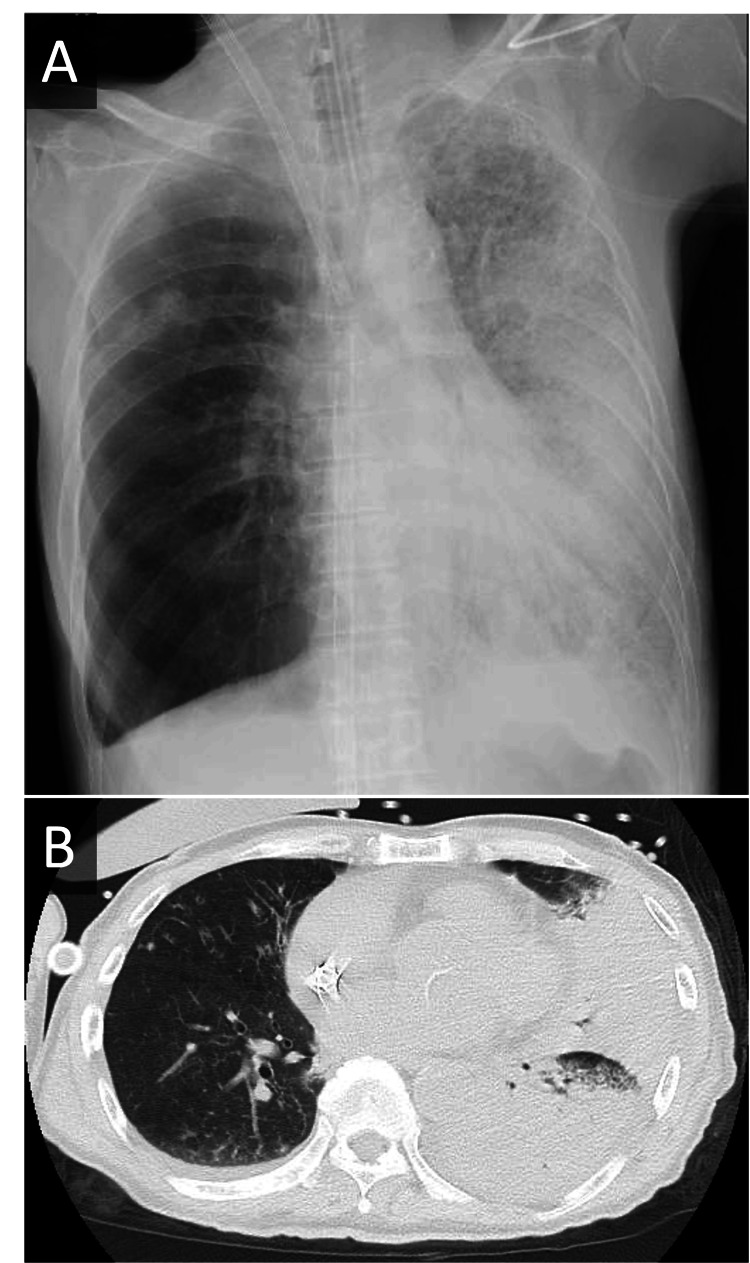
Chest Radiograph and Non-Contrast CT Scan Following Initiation of V-V ECMO (A) Chest radiograph and (B) non-contrast chest CT scan obtained after the initiation of veno-venous extracorporeal membrane oxygenation (V-V ECMO) support, demonstrating extensive infiltrative shadows throughout the left lung.

Despite initial intubation and mechanical ventilation upon arrival in the emergency room, he exhibited a progressive respiratory deterioration, accompanied by a substantial decrease in the partial pressure of oxygen in arterial blood/fraction of inspired oxygen (PaO_2_/FiO_2_) ratio to 55, leading to our decision to initiate veno-venous extracorporeal membrane oxygenation (V-V ECMO) support. His chest radiograph and non-contrast chest CT scan revealed decreased permeability and extensive infiltrative shadows in the left lung, respectively (see Figure 1). His *Legionella* urinary antigen test (Ribotest® Legionella, serogroups 1-15 detection kit; Asahi Kasei Pharma Corporation, Tokyo, Japan) and the pneumococcal urinary antigen test yielded positive results. A Gram stain of the sputum revealed numerous Gram-negative rods in the cells, along with trace amounts of Gram-positive diplococci. An HIV antibody test was negative. Given these results, the diagnosis of pneumonia due to *L. pneumophila* and *S. pneumoniae* was established, and the antibiotic treatment was escalated to meropenem, vancomycin, and levofloxacin. Baricitinib was discontinued due to concerns about worsening bacterial infections, and dexamethasone was replaced with hydrocortisone for the management of septic shock, with the aim of providing anti-inflammatory effects and improving hemodynamic stability.

**Figure 2:**
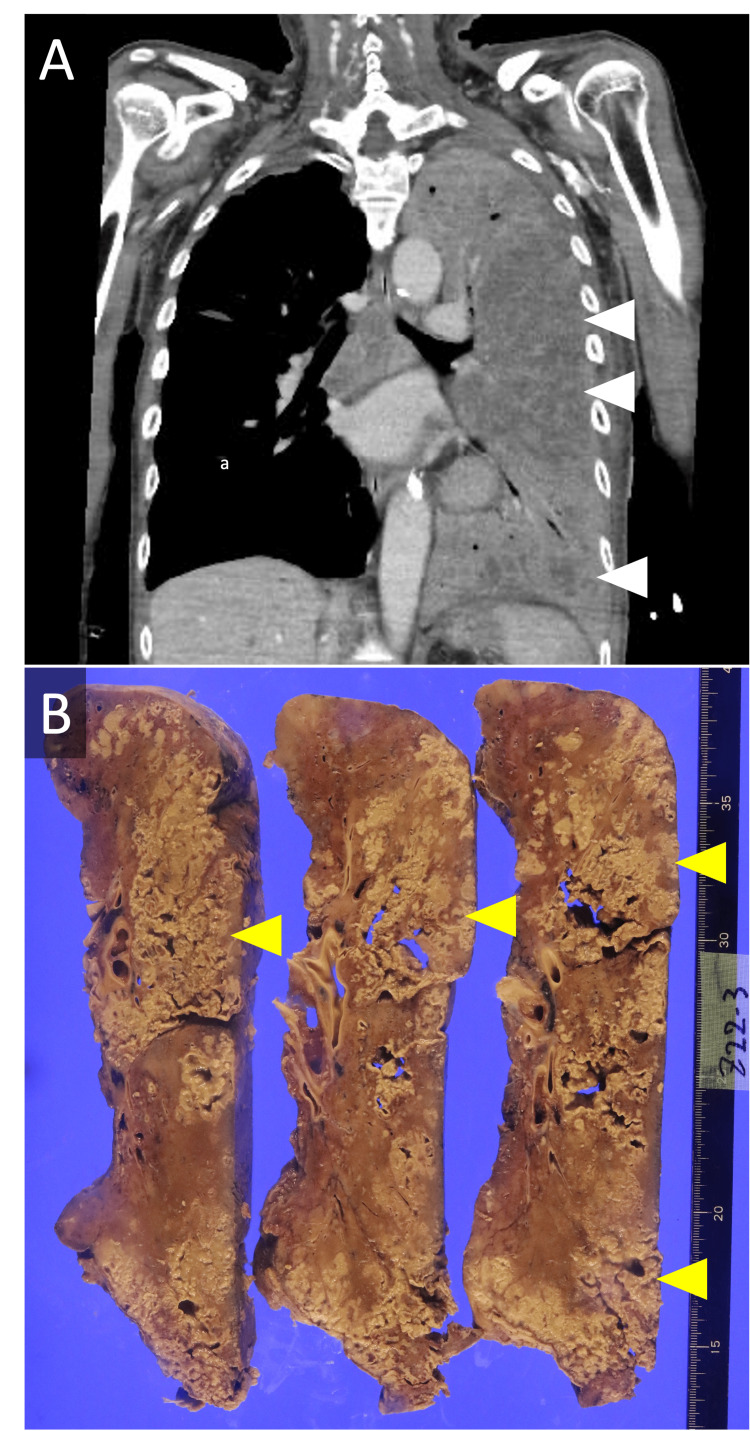
Contrast Chest CT Scan and Sectioned Specimen Showing Necrotic Tissue in the Left Lung (A) Contrast-enhanced chest CT scan taken on day 10 of his hospitalization at our hospital, showing a poorly contrasted area within the infiltrative shadow of the left lung (white arrows). (B) Sectioned specimen revealing whitish necrotic tissue (yellow arrow), corresponding to the poorly contrasted region observed on the CT scan.

On the seventh day in our hospital, a multiplex polymerase chain reaction assay (the BioFire® FilmArray® Pneumonia Panel; bioMérieux Japan, Tokyo, Japan) of his sputum collected at the time of admission revealed only *L. pneumophila* and *S. pneumoniae*. *S. pneumoniae* detected by the BioFire® FilmArray® Pneumonia Panel showed a semiquantitative value of ≥10⁷ genomic copies/mL, which strongly suggested a high bacterial load. As a result, antibiotic therapy was adjusted to ceftriaxone and levofloxacin, and azithromycin was added on the eighth day of hospitalization. A follow-up contrast-enhanced CT scan suggested the presence of necrotizing pneumonia in the dorsal side of the left lung (see Figure 2A), and the treatment for pneumonia caused by *L. pneumophila* was deemed insufficient. Therefore, rifampicin was added to the treatment regimen on the 11th day, along with ongoing prone positioning and bronchoscopic sputum evacuation. However, these interventions did not yield the anticipated positive response, and he ultimately succumbed to the infection on day 20 of his admission to our hospital.

Autopsy was conducted with the consent of his family, and the gross image of the left lung revealed multiple broad necrotic tissue areas (see Figure 2B), which corresponded to the poor-contrast regions observed on the patient’s CT scan (see Figure 2A). The necrotic tissue showed numerous neutrophilic infiltrates and microthrombi, findings consistent with a diagnosis of necrotizing pneumonia. Furthermore, the culture examination of the necrotic tissue revealed *L. pneumophila* with no agglutination by the *Legionella* agglutination test for serogroup 1-6. This finding, along with his *Legionella* urinary antigen test result, confirmed the pathogenicity of the bacterium to be *L. pneumophila* of serogroup 7-15.

## Discussion

We report an extremely rare case of concomitant *L. pneumophila* and *S. pneumoniae* infections in the context of immunosuppressive therapy for COVID-19, which unfortunately resulted in death due to severe necrotizing pneumonia refractory to appropriate antibiotic treatment. This case offers a valuable opportunity to explore the clinical implications of concomitant bacterial infections associated with immunosuppressive therapy for patients with COVID-19. It also facilitates a discussion of the diagnostic and therapeutic strategies for necrotizing pneumonia based on published case reports and various relevant studies.

Although multiple reports have addressed the potential risk of immunosuppressive agents used in the management of COVID-19, it is crucial to appropriately recognize the target patient populations in the control and intervention groups of each study when interpreting and applying the results. A retrospective study involving hospitalized patients with COVID-19 found that the concomitant use of dexamethasone and baricitinib did not increase the incidence of bacterial infections compared to dexamethasone administered as a monotherapy [4]. However, it is important to note that the mean age of the target patients in this study was 59.9 years and thus it would be inappropriate to extrapolate the findings to elderly patients. Secondly, a randomized controlled trial, which showed no significant difference in the 60-day mortality rate between baricitinib and placebo treatment groups in patients with severe COVID-19, revealed a significantly higher incidence of respiratory complications and septic shock in the baricitinib treatment group in a subgroup analysis limited to patients who were vaccinated [5]. The authors of the study hypothesized that the observed outcomes could be attributed to the fact that the vaccinated patients were older and had more comorbidities than the unvaccinated patients. The findings of the aforementioned studies suggest a plausible association between the administration of baricitinib to elderly patients with COVID-19 and increased risk of concomitant bacterial infections, as evidenced by the present case. The occurrence of the co-infections in the present case supports the hypothesis that baricitinib administration for COVID-19 may increase the risk of concomitant bacterial pneumonia and its deteriorated clinical course, particularly in elderly patients with comorbidities.

Necrotizing pneumonia is characterized by the formation of thrombus in the pulmonary microvasculature, which can impede antibiotic delivery to the infection site due to interrupted blood supply, rendering some cases medically unsalvageable [6]. In the present case, it was hypothesized that the administered antibiotics were not delivered to the necrotic tissue and thus were ineffective, although they were found to be sensitive to *L. pneumophila*, as demonstrated by the autopsy tissue results. The management of necrotizing pneumonia remains challenging, as evidenced by the absence of established guidelines. However, surgical resection of lung necrotizing lesions, including the parenchyma not enhanced in the contrast-enhanced CT scans, has been reported to be effective [7]. A case report of a young patient with respiratory failure requiring V-V ECMO for necrotizing pneumonia documented a successful outcome following lung lesion resection [8]. In light of these published reports and our present case, the necessity of early and comprehensive evaluation of necrotizing pneumonia through imaging studies followed by timely surgical intervention is crucial for a successful treatment of patients with necrotizing pneumonia, provided they are considered fit to tolerate the procedure itself.

## Conclusions

We encountered a rare case of treatment-resistant necrotizing pneumonia resulting from concomitant infections with *L. pneumophila* and *S. pneumoniae* in an elderly patient under treatment for COVID-19 with dexamethasone and baricitinib. In elderly patients with underlying comorbidities, the use of immunosuppressive agents for COVID-19 may increase the risk of bacterial co-infections with multiple pathogens. Therefore, careful consideration should be given before initiating immunosuppressive therapy for COVID-19, especially in elderly patients with comorbidities.
